# Lattice dynamics and carrier recombination in GaAs/GaAsBi nanowires

**DOI:** 10.1038/s41598-023-40217-2

**Published:** 2023-08-08

**Authors:** M. Jansson, V. V. Nosenko, G. Yu Rudko, F. Ishikawa, W. M. Chen, I. A. Buyanova

**Affiliations:** 1 Department of Physics, Chemistry and Biology, Linköping University, 58183 Linköping, Sweden; 2 Research Center for Integrated Quantum Electronics, Hokkaido University, Sapporo, 060-8628 Japan

**Keywords:** Nanoscience and technology, Optics and photonics, Physics

## Abstract

GaAsBi nanowires represent a novel and promising material platform for future nano-photonics. However, the growth of high-quality GaAsBi nanowires and GaAsBi alloy is still a challenge due to a large miscibility gap between GaAs and GaBi. In this work we investigate effects of Bi incorporation on lattice dynamics and carrier recombination processes in GaAs/GaAsBi core/shell nanowires grown by molecular-beam epitaxy. By employing photoluminescence (PL), PL excitation, and Raman scattering spectroscopies complemented by scanning electron microscopy, we show that increasing Bi-beam equivalent pressure (BEP) during the growth does not necessarily result in a higher alloy composition but largely affects the carrier localization in GaAsBi. Specifically, it is found that under high BEP, bismuth tends either to be expelled from a nanowire shell towards its surface or to form larger clusters within the GaAsBi shell. Due to these two processes the bandgap of the Bi-containing shell remains practically independent of the Bi BEP, while the emission spectra of the NWs experience a significant red shift under increased Bi supply as a result of the localization effect.

## Introduction

The search for new materials suitable for efficient near-infrared optoelectronic devices has identified dilute bismide III-V-Bi alloys as a promising candidate^1^, owing to the flexibility in tailoring numerous electronic properties of these materials upon Bi incorporation. Hybridization of the extended p-like valence-band states of the host semiconductor, e.g. GaAs, with localized p-like states of Bi leads to a strong nonlinear upshift of the valence-band edge of the forming alloy. This results in a significant reduction of its bandgap energy (E_g_)^1,2^, which could be as high as 80–90 meV/Bi% in the case of GaAsBi^3,4^, allowing efficient tuning of the alloy bandgap towards the fiber-optics communication windows of 1.3–1.55 μm. Another appealing consequence of Bi incorporation is a substantial enhancement of spin–orbit (SO) interaction, which causes a superlinear increase of the SO splitting with increasing Bi content^5^. At Bi concentrations of about 10%, the energy gap between the SO subband and valence band-edge reaches the E_g_ value of the material^6^. This should lead to suppression of nonradiative Auger recombination^6–8^, critical for the development of highly efficient near-infrared lasers emitting at the telecom wavelengths. Elimination of the non-radiative Auger recombination should also ensure reduced heat generation^2^, which is of great significance for photovoltaic and optoelectronic devices. Together with a relatively temperature-insensitive band gap of GaAsBi alloys^9–11^, these features make GaAsBi promising for devices with improved temperature stability.

However, growth of high-quality GaAsBi alloys still remains a challenge because Bi desorbs from the growing surface at growth temperatures (T_g_) that are typical for epitaxy of III–V compounds. To circumvent this problem, GaAsBi epilayers are usually fabricated under non-equilibrium growth conditions at T_g_ < 400 °C^3,12^, which leads to spatial fluctuations of Bi concentration and, therefore, local changes of the material’s bandgap^13^. Moreover, because of a much larger size of Bi atoms as compared with As atoms, the obtained alloy is highly mismatched and experiences inherent inner strain. Lattice relaxation may cause clustering of Bi atoms^14,15^, as well as their expulsion from bulk to the surface of the material^16,17^. Bi clusters induce localized states within the alloy bandgap that are located close to the valence band edge, which further contributes to the disorder-related broadening of the density of states^18^ and affects carrier recombination^19^.

Continuous miniaturization of electronic and optical devices demands utilization of nanoscale semiconductor structures, such as nanowires (NWs), with a high crystallographic and optical quality. Considering that mechanisms for the NW growth differ from that for planar structures, this poses further challenges but also new opportunities for employing highly mismatched alloys, such as GaAsBi, in nanoscale devices. Nonetheless, the first GaAsBi NWs were recently fabricated^20–26^. Similar to the planar growth of GaAsBi epilayers, bismuth was found to act not only as a constituent but also as a surfactant during the NW growth. This promotes changes of the crystal structure of thin GaAs NWs from wurtzite (WZ) to zinc blende (ZB) upon Bi presence^20^ and also leads to branching in ZB GaAs/GaAsBi core/shell NWs^25^. When incorporated, some of Bi atoms were shown to spontaneously segregate at twin planes forming optically active quantum dots^27^. In addition, a red shift of the absorption edge^28^, indicative for the alloy formation, and a red shift of the emission spectra accompanied by a broadening of the emission linewidth were reported^21,29^. In the case of thin WZ GaAsBi NWs with very low Bi compositions^29^, these changes in the emission spectra were attributed to trapping of excitons within band-tail states. On the other hand, effects of carrier localization in ZB GaAsBi NWs were not carefully studied so far, even though one may expect that they should be severe in these highly mismatched structures. In the present study we clarify this important issue based on systematic optical studies employing micro photoluminescence (µ-PL) spectroscopy complemented by µ-PL excitation (µ-PLE) and Raman measurements.

## Samples and methods

**Figure 1:**
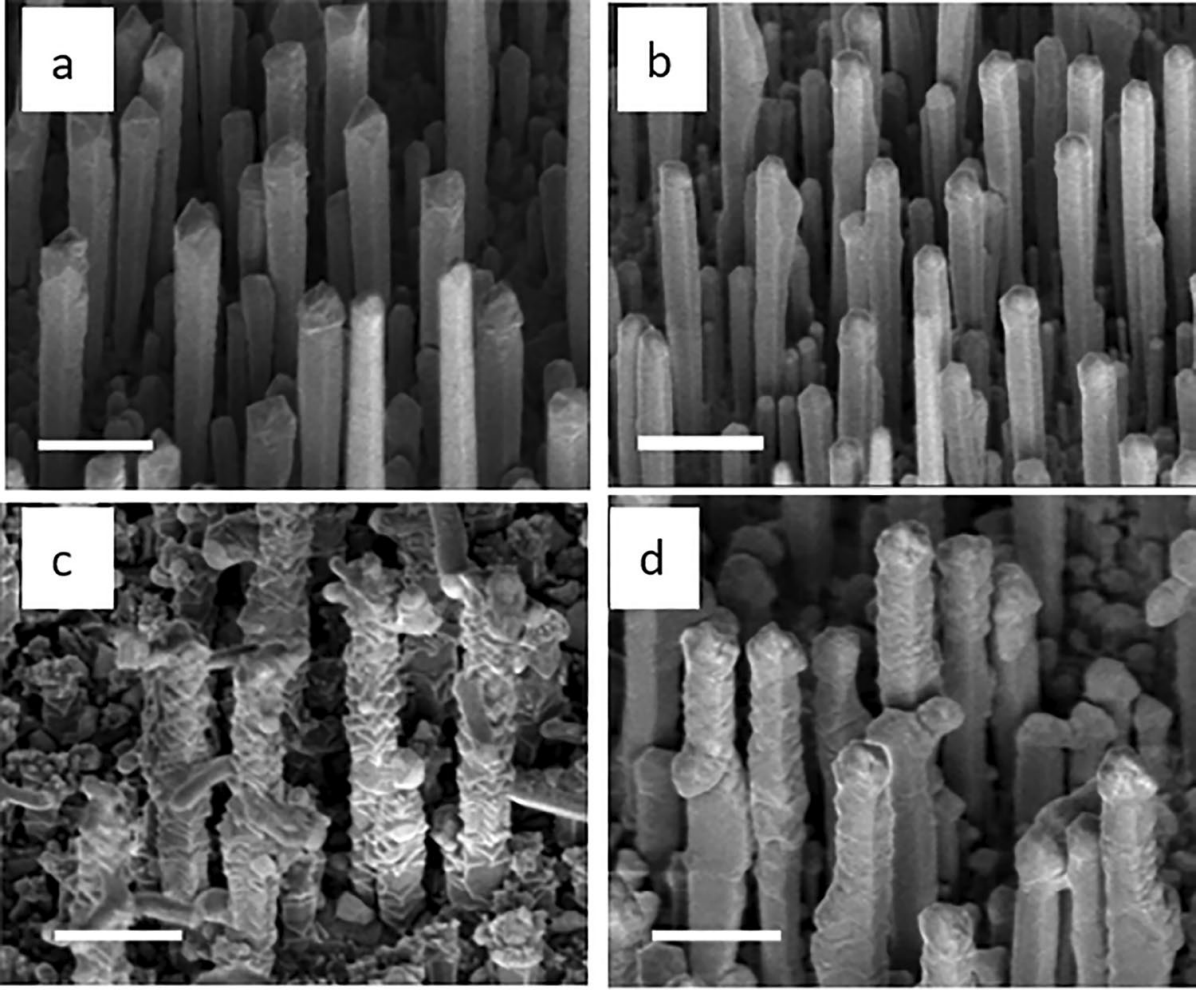
SEM images of the NW arrays: (**a**) GaAs NWs; (**b**) low BEP GaAs/GaAsBi core/shell NWs; (**c**) high BEP GaAs/GaAsBi core/shell NWs; (**d**) GaAs/GaAsBi/GaAs CSS structures. The scale bar is 1 µm.

The investigated GaAs/GaAsBi core/shell NWs were grown on (111) Si substrates using solid-source molecular beam epitaxy (MBE). The details of the growth process can be found elsewhere^21,22,25,30^. To initiate vapor–liquid–solid growth catalyzed by Ga droplets, the GaAs core was grown at 580 °C under As_4_ overpressure with the As_4_ beam equivalent pressure (BEP) of ~ 1 × 10^−3^ Pa. The Ga beam flux was set to match the planar growth rate of 1.0 ML/s on a GaAs (001) substrate. After the crystallization of the catalyst, lateral growth of the GaAsBi shell was performed via vapor–solid process under fixed Bi BEP. To facilitate Bi incorporation, the shell growth was performed at a low growth temperature of 350 °C. The following four types of NW structures were studied: (i) GaAs/GaAsBi core/shell NWs with the GaAsBi shell grown at Bi BEP of 6 × 10^−6^ Pa (to be referred to below as low-BEP NWs); (ii) GaAs/GaAsBi core/shell NWs with the shell grown under Bi BEP of 5.4 × 10^−5^ Pa (to be refereed to below as high-BEP NWs); (iii) GaAs/GaAsBi/GaAs core/shell/shell (CSS) NWs with the GaAsBi inner shell grown under high BEP of 5.4 × 10^−5^ Pa (to be referred to below as CSS NWs); and (iv) pure GaAs NWs serving as a reference. The Bi BEP values were chosen to obtain targeted Bi concentrations of approximately 1.3% and 2% in the GaAsBi shell^21,22,25,30^. The deposition of an outer shell in the CSS structure was done to passivate the surface of the optically-active GaBiAs shell, as has been demonstrated successfully in many other material systems^31,32^. Typical scanning electron microscopy (SEM) images of the investigated NW structures are shown in Fig. 1. In all cases, the NWs are found to form dense arrays of vertically aligned NWs with the length varying between 1 and 6 μm. The NW morphology, however, depends on the Bi BEP during the growth. Whereas the low-BEP NWs shown in Fig. 1b have a smooth surface morphology and sharp facets that are similar to the reference structures shown in Fig. 1a, the high-BEP NWs demonstrate large surface corrugations and roughening accompanied by formation of side branches. The branching is suppressed in the core/shell/shell structures shown in Fig. 1d. These changes in NW morphology reflect complex effects of Bi on the NW growth as was discussed in detail in Refs.^21,25,27,30^. According to the performed transmission microscopy measurements, all NWs have ZB crystal structure with minor WZ inclusions^27,30^.

Optical characterization was carried out using a micro-PL/Raman setup. The micro-PL/Raman system was set up by focusing the excitation light using a microscope objective with 50× magnification and 0.5 numerical aperture. Therefore, a spot size of approximately 1 µm in diameter can be achieved allowing investigations of single NWs. The same objective lens was also used to collect the emitted/scattered light from the sample, which was then filtered by appropriate long-pass (edge) filters for PL and PLE (Raman) measurements, and subsequently dispersed by a single-grating monochromator and registered by a LN_2_-cooled InGaAs linear array detector. The micro-PL system was supplied by Horiba. As an excitation source, we used a 660 nm solid state laser for PL and Raman measurements and a tunable continuous-wave Ti:sapphire laser for PLE measurements. Temperature dependent measurements in the range of 7–300 K were conducted by mounting the samples in a Microstat HiRes cryostat from Oxford Instruments.

## Experimental results and discussion

### Raman spectroscopy

**Figure 2:**
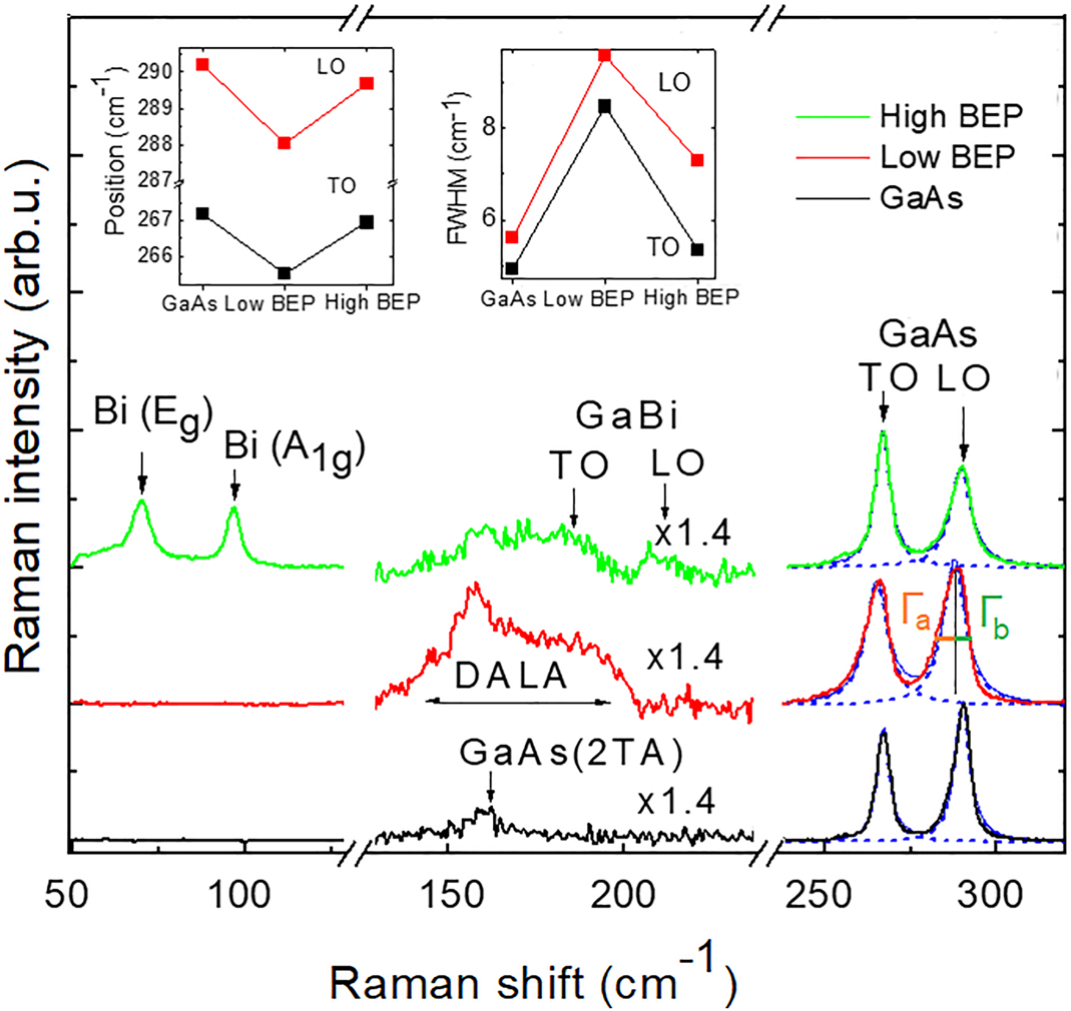
Raman spectra of the GaAs and GaAs/GaAsBi NWs measured at room temperature. The left and right insets show variations of the spectral positions of the LO and TO modes and their FWHM, respectively, between the different NWs.

Effects of Bi incorporation on lattice dynamics in the studied structures are evaluated using Raman spectroscopy. The corresponding results are summarized in Fig. 2, which shows Raman spectra of the NW arrays measured at room temperature in the 50–325 cm^−1^ spectral range. The Raman spectrum of the GaAs NWs contains two dominant peaks at 267 cm^−1^ and 290 cm^−1^ due to one-phonon scattering of zone-center transversal optical (TO) and longitudinal optical (LO) phonons, respectively^33–35^. In addition, a weak mode at 161 cm^−1^ associated with two-photon scattering involving zone-edge transverse acoustic (2TA) phonons^35^ is also observed. The detected Raman spectrum confirms the ZB crystalline structure of the studied NWs, since an additional E_2_^h^(TO) mode at around 256 cm^−1^ should be detected in WZ GaAs, due to folding of phonon dispersion along the [111] (Γ → L) direction^33,36^.

Bi presence leads to several modifications of the Raman spectra. First of all, it causes an appearance of two weak Raman peaks at 185 and 210 cm^−1^. According to previous studies^37–39^, these peaks correspond to the TO and LO vibrational modes of GaBi. The presence of GaBi-like TO and LO modes verifies the intended incorporation of Bi into the GaAs lattice in both types of NWs. As expected, the intensity of the GaBi phonons is significantly weaker than that of the GaAs modes, consistent with the low concentration of Bi in the alloy. Secondly, Raman spectra of the GaAsBi NWs contain a broad band in the spectral range of 130–200 cm^−1^, which corresponds to the disorder-activated longitudinal acoustic (DALA) phonons^40^. Additionally, the Raman spectrum of high-BEP NWs demonstrates two low frequency peaks at 70 cm^−1^ and 96 cm^−1^. The energy positions of these modes coincide with the known E_g_ and A_1g_ modes of metallic Bi^41–43^, implying that some Bi atoms in the high-BEP NWs are not incorporated into GaAs but rather form metal droplets on the NWs surface, consistent with^16,17^. Finally, alloying with Bi leads to a red shift of the dominant GaAs-like LO and TO modes accompanied by broadening and asymmetry of these Raman lines. Changes of the Raman lineshape are indicative of alloy disorder^44^, whereas the red shift of the GaAs-like modes reflects combined effects of alloying, strain and disorder^39,44^. Indeed, according to the spatial phonon correlation model^44^, the alloy potential fluctuations and related degradation of long-range order lead to the relaxation of the *q* = 0 momentum selection rules^44,45^. The *q* ≠ 0 transitions contribute to the Raman spectrum at energies that are determined by the phonon dispersion. Since optical phonons in GaAs have the largest energy at the Γ-point, relaxation of the selection rules causes a red shift of the maximum position of these Raman modes and their asymmetric broadening towards lower energies.

Considering that increasing BEP during the growth is intended to increase the alloy content, one would expect a monotonous increase of the red shift, broadening and asymmetry of the Raman lines under these conditions, as was observed previously in GaNAs NWs^46^. Surprisingly, this is not the case. This can clearly be seen from the insets in Fig. 2, where the peak positions (the left inset) and the full width at half maximum (FWHM) (the right inset) of the GaAs-like modes are shown. To further visualize the line asymmetry, we have performed fitting of these Raman peaks using a Lorentzian function. Moreover, their FWHM was divided into the low energy (Γ_a_) and high energy (Γ_b_) parts, as shown in Fig. 2. It is noticeable that whereas the Lorentzian function with Γ_a_ = Γ_b_ satisfactorily describes the lineshape of the TO and LO modes in GaAs NWs, this is no longer the case in Bi-containing structures, where Γ_a_ is larger than Γ_b_. We find that for the high-BEP NWs the Γ_a_/Γ_b_ ratio is equal to 1.25 (or 1.26) for the TO (or LO) mode, while the strongest asymmetry of the lineshape is observed in the spectrum of the low-BEP NWs, where Γ_a_/Γ_b_ = 1.44 for the TO mode and 1.69 for the LO mode. Based on the phonon correlation model^44^, this translates to the phonon correlation lengths of about 63 nm and 110 nm for the low-BEP and high-BEP NWs, respectively. Considering that the phonon correlation length in ternary alloys reflects an extent to which the sublattice is ordered, this in turn implies the highest degree of disorder in the NWs grown with the low Bi BEP, consistent with a higher intensity of the DALA mode in this structure. We will return to this unusual result later after analyzing results of the PL measurements.

### PL and PLE spectroscopy

**Figure 3:**
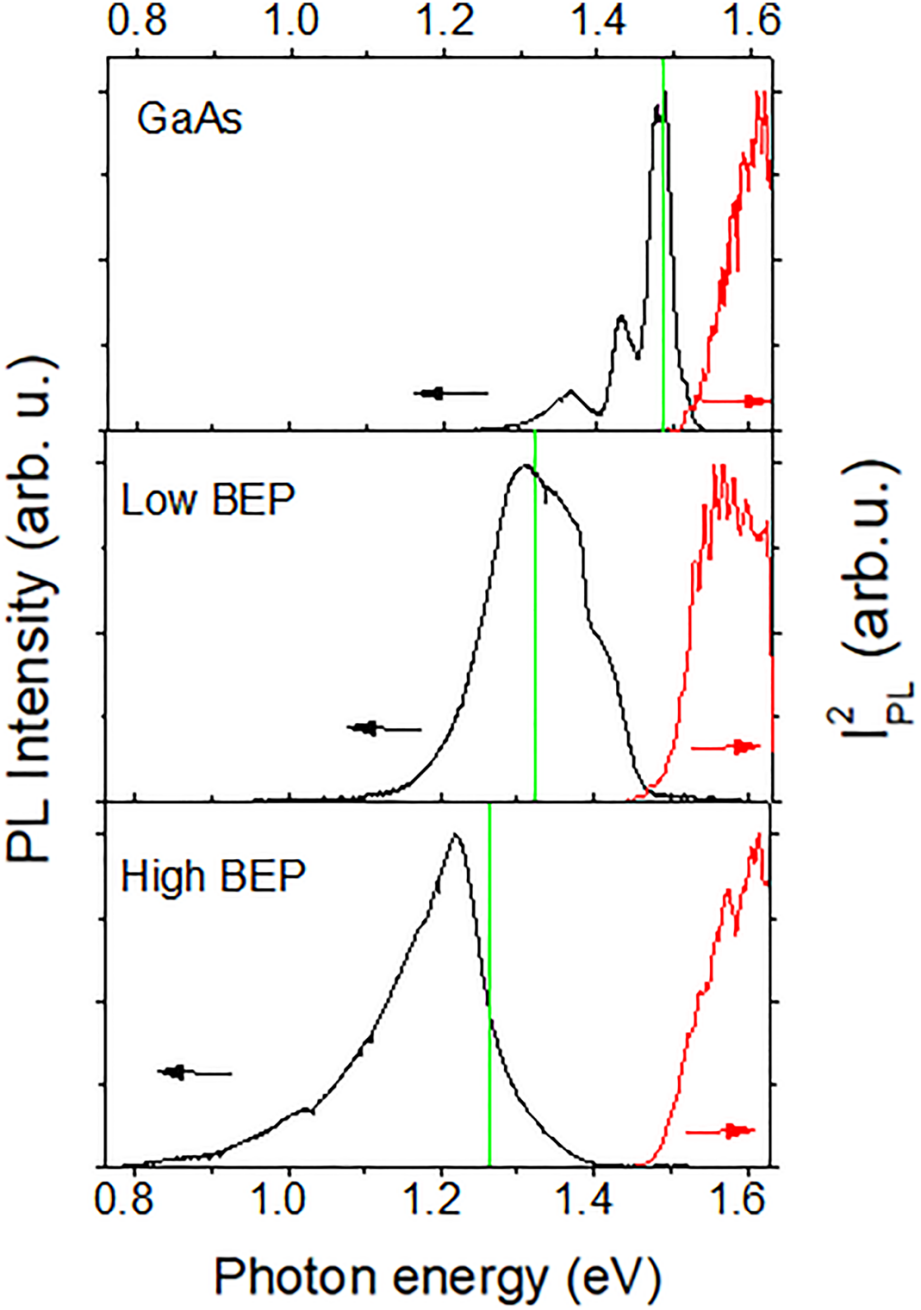
The PL (the black curves) and PLE (the red curves) spectra measured at 6 K from the investigated GaAs/GaAsBi NWs. The detection energies during the PLE measurements are indicated by the vertical green lines. In order to extrapolate the bandgap energy, the PLE spectra are displayed as the square of the PL intensity (I_PL_).

To further characterize the alloy disorder and Bi-induced electronic states in the studied structures, the PL and PLE measurements were performed—see Fig. 3. The PL spectra of the GaAs NWs are found to be typical for such structures and include the dominant PL band peaking at around 1.494 eV due to free-to-bound transitions involving carbon acceptors, as well as a weak free exciton emission at a higher energy. On the other hand, the PL spectra of the Bi-containing NWs are dominated by a broad emission band, which experiences a significant red shift by ∼106 meV with increasing Bi BEP (see the black curves in Fig. 3). Though it could be natural to assume that the observed red shift stems from a gradual reduction of the bandgap energy in GaAsBi alloys, the results of the performed PLE measurements show that this is not the case. This is because the onsets of the PLE spectra are found to occur almost at the same energy in both Bi-containing NWs (the red curves in Fig. 3). Since a PLE spectrum usually reflects absorption transitions involving extended band states, its onset is determined by the bandgap energy of the emitting material. Therefore, to determine bandgap energies of the studied NWs, we linearly extrapolate the dependence of the squared PL intensity as a function of excitation energy. This yields E_g_ = 1.519 eV for the GaAs NWs and 1.475 eV for the high- and low-BEP structures. The results of Fig. 3 imply that though the bandgap energies and, thus, alloy compositions of GaAsBi are the same in the high- and low-BEP NWs, their emission spectra are distinctly different. This apparent contradiction between the PL and PLE data can arise due to different distributions of Bi-related localized states in the materials. Indeed, in contrast to the PLE technique that probes the band states and is generally not sensitive to the localized states, the PL spectroscopy exposes all energy states participating in radiative recombination, including localized states caused by the presence of impurities that are favorably populated at low measurement temperatures. The observed changes of the PL spectral position with increasing Bi BEP suggest that the increased Bi supply during the growth does not change the alloy composition but promotes the formation of Bi-related localized states that are located deeper within the bandgap.

**Figure 4:**
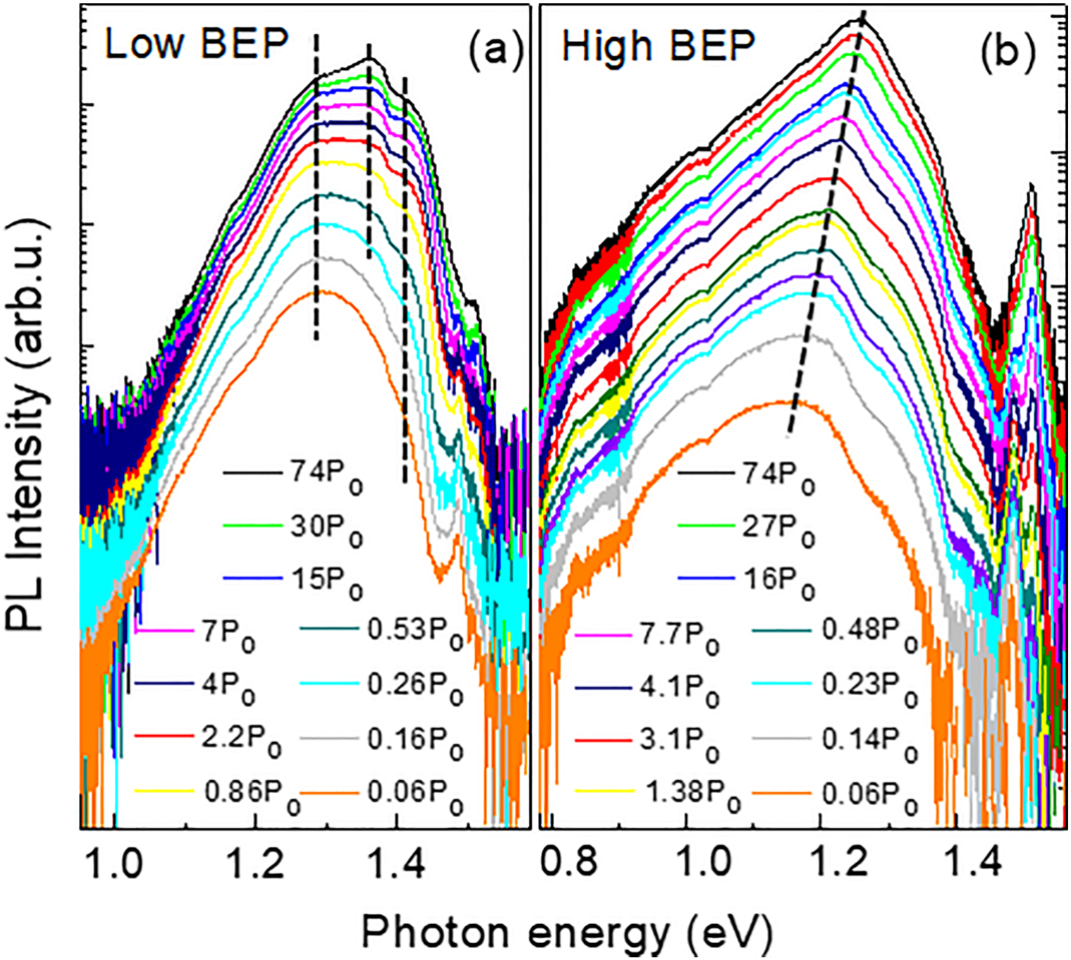
Evolution of the PL spectra measured at 6 K from the low-BEP and high-BEP NWs with increasing excitation power density (P). Here P_0_ = 50 nW μm^−2^. The weak emission seen at around 1.5 eV originates from the GaAs core.

Further information regarding the properties of these states is obtained based on power-dependent μ-PL measurements. The results of these measurements are summarized in Fig. 4. With increasing excitation power (*P*), the emission spectra in both types of NWs shift towards higher energies and exhibit a tendency to intensity saturation, which becomes especially pronounced at high *P* (see the Supporting information, Fig. S1). A more detailed analysis of the spectra, however, reveals certain differences. It becomes obvious that the PL band in the low-BEP NWs comprises of several overlapping components. The peak positions of these components remain independent of the excitation power, whereas the contribution of the high energy components becomes more pronounced at high *P*. This causes an overall blue shift of the PL spectrum—see Fig. 4a. On the other hand, such individual PL components could no longer be resolved for high-BEP NWs. Nonetheless, the intensity within this broad PL band is also redistributed in favor of the transitions with larger energies of the emitted quanta with increasing *P*, thus causing a gradual blue shift of the PL maximum position—see Fig. 4b.

The transformation of the PL spectra from a series of individual PL components to a structureless band has previously been reported in highly mismatched alloys^47^, including GaAsBi^48^. Spectrally-resolved PL features similar to the ones detected in the low-BEP NWs were observed in GaAsBi epilayers with low Bi concentrations below 0.4%^48^. They were attributed to excitons bound at localized Bi-related centers, such as pairs of Bi atoms and small Bi clusters. Increasing Bi concentration in the alloy above 0.8% facilitated formation of larger Bi clusters, which formed a band of closely-spaced energy levels located deeper within the bandgap. Consequently, only a structureless PL band at lower energies was seen. We suggest that the same mechanism of the PL emission is also applicable to the investigated structures, namely, the PL emission in the low-BEP NWs is related to Bi-pairs and small clusters whereas in the high-BEP NWs it stems from deeper localized states induced by large Bi clusters. The observed tendency of saturation of the PL emission under high excitation power further confirms this assignment, as it implies a limited density of states participating in light emitting processes, as well as a rather low recombination rate, typical for excitons bound to Bi-related centers^48^. We note, however, that the energy positions of the individual PL components in our structures differ from the ones previously detected in GaAsBi epilayers, indicating that different cluster states are formed. Moreover, our results also suggest that the evolution of the Bi-related localized states is not only linked to the alloy composition but also depends on the growth conditions, such as Bi BEP during the NW growth.

**Figure 5:**
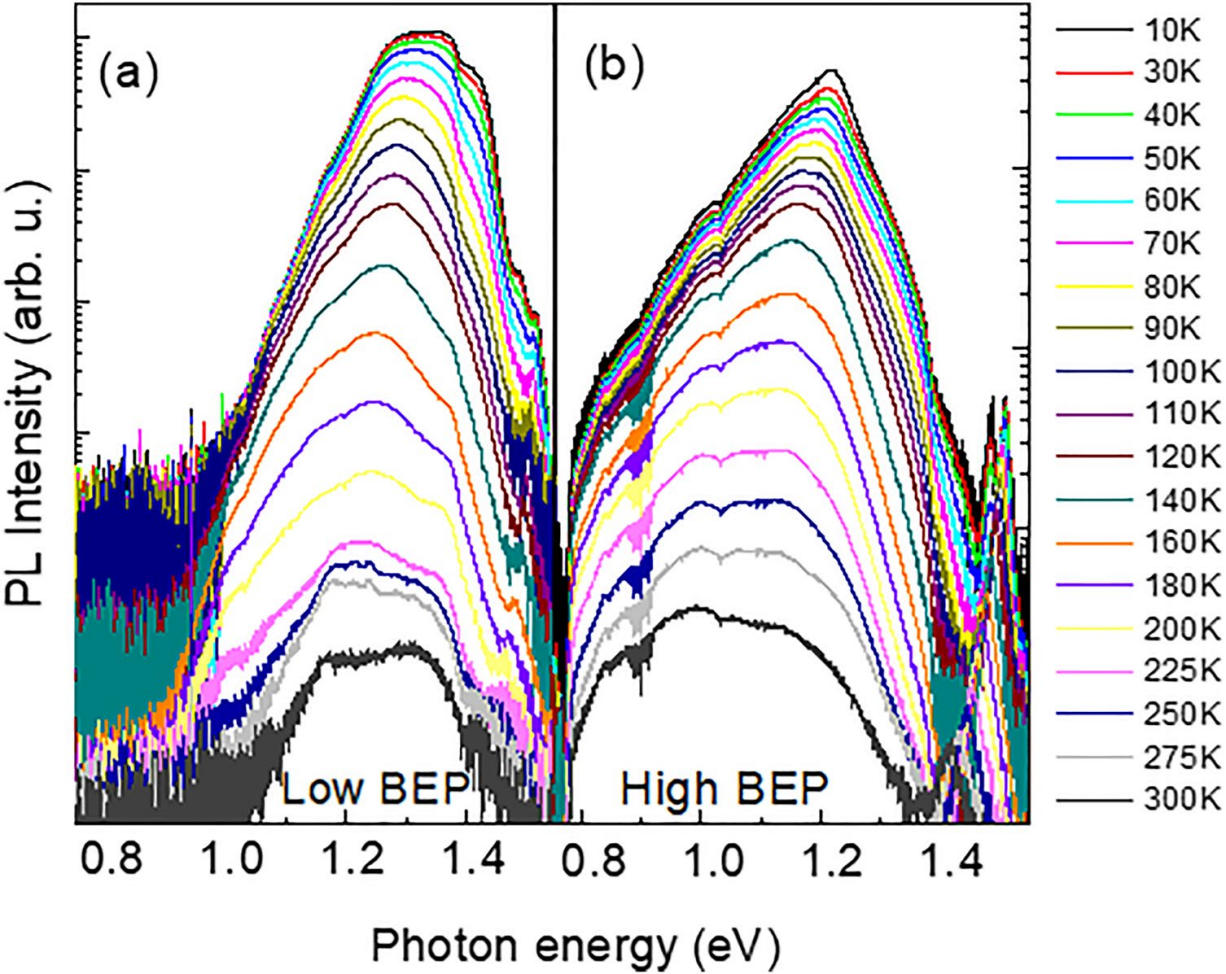
Temperature dependence of the PL spectra from the low-BEP (**a**) and high-BEP (**b**) NWs measured at the excitation power of 350 nW μm^−2^.

From Figs. 3 and 4, carrier localization significantly affects carrier recombination at low temperatures. To understand its impact at elevated temperatures relevant to device operation, we performed temperature-dependent PL studies. It is found that in both structures a temperature (T) increase causes an overall red shift of the PL spectra—see Fig. 5. In the low-BEP NWs this red shift, which is especially pronounced at T < 200 K, is caused by two contributing effects: (i) a red shift of the individual PL components, likely reflecting a down-shift of the conduction band edge with increasing temperature; and (ii) quenching of the high energy components due to thermally-activated escape of the localized excitons from the shallower Bi-related localized states and their subsequent capture by deeper localized states and non-radiative centers. Though further increase of T leads to a re-distribution of the photo-excited carriers between the localized states, no activation of the free carrier recombination at elevated temperatures is seen. This likely reflects a high density of the Bi-related localized states. Very similar thermal behavior is also seen from the high-BEP NWs, though the close energy spacing between the cluster states in these structures leads to a gradual shift of the PL maximum position with increasing T.

**Figure 6:**
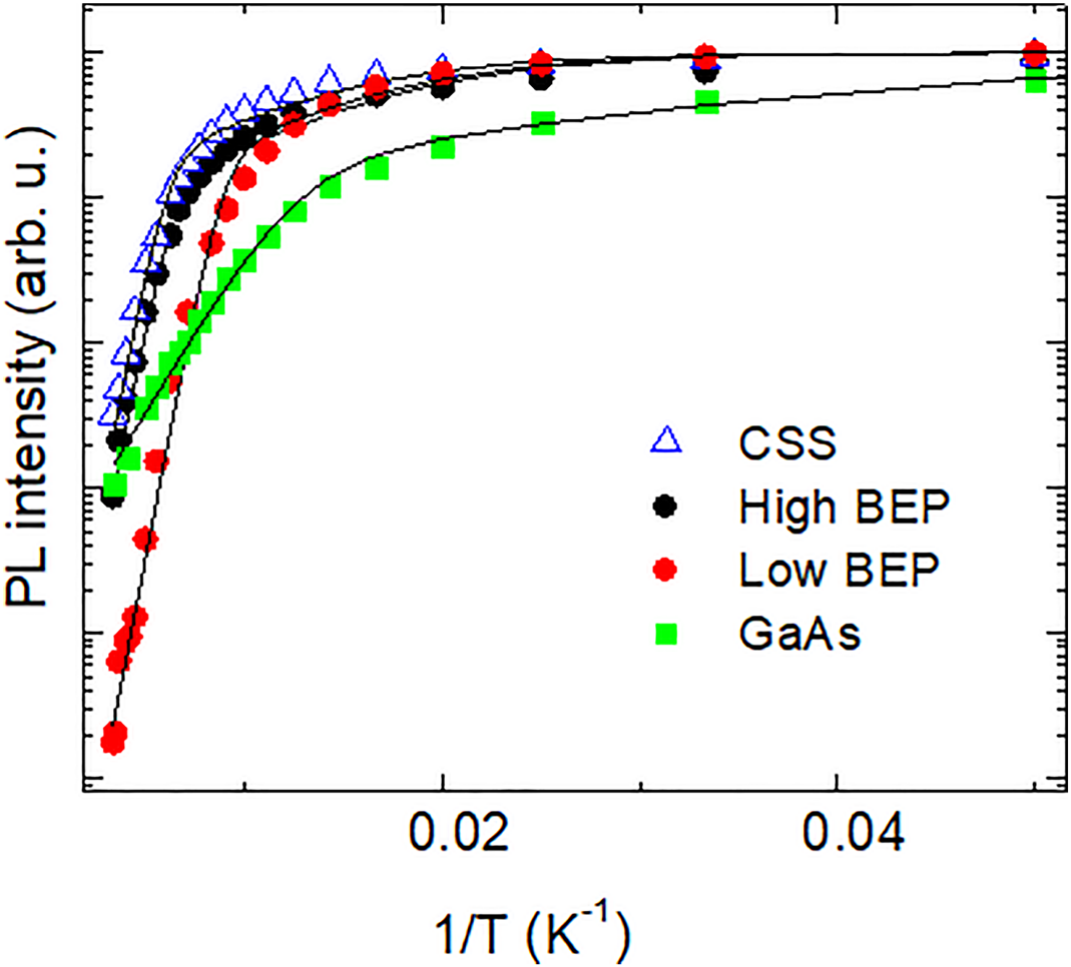
Arrhenius plots of the integrated PL intensity measured from the specified Bi-containing NWs and the reference GaAs NWs. The symbols represent the experimental data, whereas the solid lines are the best fit to the data using Eq. (1) with the fitting parameters given in Table 1.

From Fig. 5, it is apparent that an increasing temperature also leads to an overall quenching of the PL intensity in both NW structures. This degradation of internal quantum efficiency of the NWs is caused by activation of competing non-radiative (NR) recombination that limits the lifetime of photo-excited carriers. To gain further insight into effects of alloying with Bi on the non-radiative recombination processes in the studied NWs, we analyze Arrhenius plots of the integrated PL intensities measured from the Bi-free and Bi-containing structures—see Fig. 6. In all cases the temperature dependence of the PL intensity *I* can be described by the equation

1 $$I\left( T \right) = \, I\left( 0 \right)/[1 + c_{1} \exp\left( { - E_{1} /kT} \right) + c_{2} \exp( - E_{2} /kT)],$$

where k is the Boltzmann constant, and T is the temperature. Here E_1_ and E_2_ are the activation energies of the two dominant non-radiative recombination processes, whereas the corresponding pre-factors c_1_ and c_2_ reflect the relative contributions of these NR recombination. The best fits to the experimental data (symbols) by using Eq. (1) are shown by the black lines in Fig. 6, with the fitting parameters summarized in Table 1. It is found that the activation energies of the dominant NR recombination processes differ significantly between the Bi-free and Bi-containing NWs, indicating that Bi incorporation critically affects the defect formation, consistent with the previous studies of GaAsBi epilayers^49,50^. On the other hand, changing Bi BEP during the NW growth does not influence the origin of the involved defect, since the activation energies of the PL thermal quenching in the low- and high-BEP NWs remain the same. We note that the onset of the PL thermal quenching is observed at higher temperatures in the high-BEP NWs than in the low-BEP structures. This suggests a lower density of the NR recombination centers in this sample, indicating that the formation of the involved defects is affected by changes in the growth kinetics. It is known that an important source of the NR recombination in III-V NWs are surface states. In order to evaluate their contribution in the studied structures, we also study thermal quenching of the PL emission in the GaAs/GaAsBi/GaAs core/shell/shell NWs, where the surface of the GaAsBi shell grown at high Bi BEP is passivated by an outer GaAs shell. It is found that this surface passivation does not improve the PL thermal quenching, indicating that the dominant NR defects in the GaAsBi NWs are not surface-related but are formed within the bulk regions of the GaAsBi shell. (We note that general PL properties of the core/shell/shell NWs are very similar to that of the high-BEP structures, as shown in the Supporting information, Fig. S2)

.

**Table 1:** Parameters deduced from the best fit of the experimentally obtained temperature dependence of the PL intensity by Eq. (1).

Sample	E_1_, meV	c_1_	E_2_, meV	c_2_
GaAs NWs	45	4000	5	9
High BEP NWs	140	250,000	15	20
CSS NWs		100,000		11
Low BEP NWs		10,000,000		17

## Discussion

The aforementioned experimental results reveal the following trends in the Bi-containing shells of the GaAs/GaAsBi NWs with increasing Bi BEP: (i) increasing surface roughness and even branching; (ii) the formation of Bi droplets on the NW surface; (iii) on average, decreasing long-range alloy disorder within the GaAsBi shell; (iv) the formation of larger Bi clusters in the GaAsBi alloy; (v) the same alloy composition (within the range of the employed BEP). All these trends reflect drastic changes in the Bi incorporation pattern into the GaAsBi shell under increased Bi supply, which can be explained as follows.

The GaAsBi shell grown at low Bi BEP is a ternary compound with appreciable short-range disorder that is revealed by the Raman studies, demonstrating the heterogeneous broadening and shift of the GaAs-like LO and TO modes (Fig. 2). The observed disorder is related to composition fluctuations that are typical for highly mismatch alloys. Since Bi is an isoelectronic impurity in GaAs, small Bi clusters form shallow localized states in the bandgap of the material. Based on the power-dependent (Fig. 4a) and temperature-dependent (Fig. 5a) PL measurements, these small clusters introduce well-spaced energy levels within the bandgap so that the individual emissions related to these states can be resolved.

At high BEP, an increased supply of Bi atoms does not facilitate their incorporation in the GaAsBi alloy. In fact, the amount of Bi that is homogeneously distributed within the lattice of the high-BEP NW shell remains the same as the alloy composition of the GaAsBi shell in the low-BEP NWs. This is confirmed by the same spectral onsets of the PLE spectra (Fig. 3) and, therefore, the same bandgap energies of these materials. Instead, the additionally supplied Bi is either expelled from the volume forming Bi droplets on the NW surface detected by the Raman measurements (Fig. 2) or is consumed by forming large Bi clusters that introduce deep bandgap states revealed by PL. The driving force for these processes could be relaxation of strain created due to size mismatch between Bi and As atoms. The observation of nonuniform distribution of Bi is consistent with the previous thermodynamical analysis^51^, which has shown that the incorporation of Bi into the lattice becomes thermodynamically unfavorable when Bi droplets are already formed at the growing surface. As a result of the Bi redistribution, the high-BEP shell as a whole becomes less strained and more homogeneous (except for the regions of Bi clustering) as compared with that in the low-BEP NWs. The latter statement is confirmed by a smaller disorder-related shift and a decrease of the Raman lines asymmetry (Fig. 2).

## Conclusions

In summary, a range of optical techniques, including excitation power- and temperature- dependent PL, PL excitation and Raman scattering measurements, were employed to reveal effects of growth conditions on lattice dynamics and recombination processes in GaAs/GaAsBi core/shell NWs. It is shown that Bi incorporation significantly enhances the alloy disorder in the material leading to a decrease in the phonon correlation length and also formation of the Bi-related clusters governing radiative recombination in the material. It is also demonstrated that these processes are affected by the Bi supply during the growth. Specifically, it is shown that increasing the Bi BEP during the growth of the NW shell under the utilized growth conditions leads to a significant red shift of the PL emission but does not cause noticeable changes of the alloy bandgap energy. This is attributed to segregation of the additionally supplied Bi atoms inside the alloy leading to an increase in size of the formed Bi clusters, which introduce deep energy states inside the bandgap as revealed by the PL spectroscopy. According to the Raman measurements, some of the Bi atoms in high-BEP NWs are also expelled towards the surface forming Bi droplets. These insights provide a valuable guideline for future optimization of the growth processes rendering high-performance GaAsBi NW structures for near-infrared optoelectronics.

### Supplementary Information

Supplementary Figures.

## Data Availability

All data generated or analysed during this study are included in this published article [and its supplementary information file].
