# Supplementary material for: Lattice dynamics and carrier recombination in GaAs/GaAsBi nanowires
Source: Sci Rep. 2023 Aug 8;13:12880. doi: 10.1038/s41598-023-40217-2 (PMC10409742; doi:10.1038/s41598-023-40217-2)
Supplement: Supplementary file 1 — Supplementary Figures. [file 41598_2023_40217_MOESM1_ESM.pdf]

## Supporting Information for the paper

# Lattice dynamics and carrier recombination in GaAs/GaAsBi nanowires

M. Jansson[1], V.V. Nosenko[1], G.Yu. Rudko[1], F. Ishikawa[2], W.M. Chen[1], and I.A. Buyanova[1]

[1]*Department of Physics, Chemistry and Biology, Linköping University, 58183 Linköping, Sweden*

[2] *Research Center for Integrated Quantum Electronics, Hokkaido University, Sapporo 060-8628, Japan*

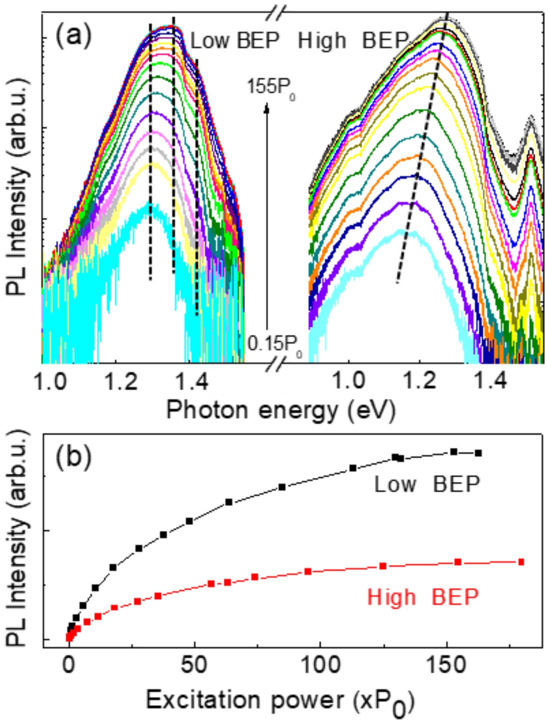


Figure S1. (a) Evolution of the PL spectra measured at 4 K from the low-BEP and high-BEP NWs with increasing excitation power density (P). The measurements were performed using a frequency-doubled pulsed Ti:sapphire laser at 770 nm with a pulse width of 150 fs. (b) The PL intensity of the low-BEP and high-BEP NWs as a function of the excitation power. Here $P_0$ = 0.39 μJ/cm$^2$/pulse.

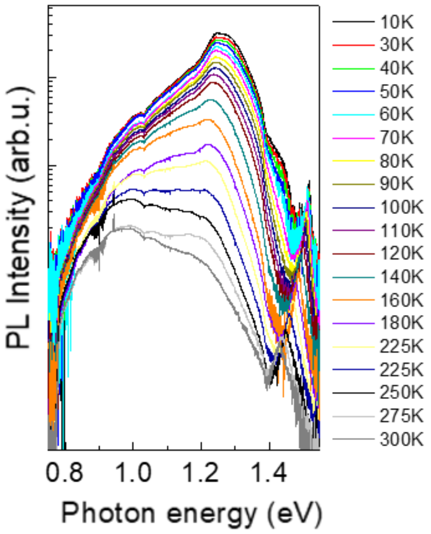


Figure S2. Temperature dependence of the PL spectra measured from the GaAs/GaAsBi/GaAs core/shell/shell NWs under the excitation photon power of 350 nW/μm$^2$.
